# TRAP1 Controls Mitochondrial Fusion/Fission Balance through Drp1 and Mff Expression

**DOI:** 10.1371/journal.pone.0051912

**Published:** 2012-12-20

**Authors:** Hironori Takamura, Yoshihisa Koyama, Shinsuke Matsuzaki, Kohei Yamada, Tsuyoshi Hattori, Shingo Miyata, Kana Takemoto, Masaya Tohyama, Taiichi Katayama

**Affiliations:** 1 Molecular Research Center for Children’s Mental Development, United Graduate School of Child Development, Osaka University, Suita, Osaka, Japan; 2 Department of Child Development and Molecular Brain Science, United Graduate School of Child Development, Osaka University, Suita, Osaka, Japan; 3 Department of Anatomy and Neuroscience, Graduate School of Medicine, Osaka University, Suita, Osaka, Japan; 4 Department of Molecular Neuropsychiatry, Graduate School of Medicine, Osaka University, Suita, Osaka, Japan; 5 Division of Molecular Brain Science, Research Institute of Traditional Oriental Medicine, Kinki University, Sayama, Osaka, Japan; University of Sheffield - MRC Centre for Developmental and Biomedical Genetics, United Kingdom

## Abstract

Mitochondria are dynamic organelles that change in response to extracellular stimuli. These changes are essential for normal mitochondrial/cellular function and are controlled by a tight balance between two antagonistic pathways that promote fusion and fission. Although some molecules have been identified to mediate the mitochondrial fusion and fission process, the underlying mechanisms remain unclear. Tumor necrosis factor receptor-associated protein 1 (TRAP1) is a mitochondrial molecule that regulates a variety of mitochondrial functions. Here, we examined the role of TRAP1 in the regulation of morphology. Stable TRAP1 knockdown cells showed abnormal mitochondrial morphology, and we observed significant decreases in dynamin-related protein 1 (Drp1) and mitochondrial fission factor (Mff), mitochondrial fission proteins. Similar results were obtained by transient knockdown of TRAP1 in two different cell lines, SH-SY5Y neuroblastoma cells and KNS-42 glioma cells. However, TRAP1 knockdown did not affect expression levels of fusion proteins. The reduction in Drp1 and Mff protein levels was rescued following treatment with the proteasome inhibitor MG132. These results suggest that TRAP1 regulates the expression of fission proteins and controls mitochondrial fusion/fission, which affects mitochondrial/cellular function.

## Introduction

Mitochondrial morphology is regulated by continuous fusion and fission to form highly connected networks or fragmented units. These dynamic changes are necessary for normal mitochondrial and cellular functions [1]. Some molecules have been reported to be key regulators of these changes. Mitofusin (Mfn) 1/2 and optic atrophy 1 (OPA1) are important for mitochondrial fusion, while dynamin-related protein 1 (Drp1), fission 1 (Fis1) and mitochondrial fission factor (Mff) are important for mitochondrial fission [2]–[9]. Drp1 localizes mainly in the cytoplasm, and a small amount of Drp1 localizes to the mitochondria representing future fission sites [6], [10]. In yeast, Fis1 induces mitochondrial fragmentation, and down-regulation of Fis1 induces elongated mitochondria. Furthermore, Drp1 and Fis1 coimmunoprecipitate after cross-linking in vitro, suggesting that mitochondrial fission mechanisms are somewhat conserved throughout eukaryotes [9]. In mammals, Drp1 physiologically interacts with Mff. Mff is an essential factor for mitochondrial recruitment of Drp1 during mitochondrial fission in mammalian cells [5]. Morphological changes of mitochondria are closely associated with apoptosis, and Drp1 is essential for the normal progression of apoptosis [11]–[15]. Apoptotic stimuli trigger mitochondrial fission, cristae disorganization, permeabilization of the mitochondrial outer membrane, and release of apoptosis regulatory proteins, including cytochrome c [16], [17]. In addition, recent studies suggest that mitochondrial fission is involved in the degradation of mitochondria via autophagy (mitophagy) [18], [19]. Abnormal mitochondrial dynamics often cause neuronal synaptic loss and cell death in several human neurodegenerative diseases, such as Alzheimer’s disease, Parkinson’s disease and Huntington’s disease [20]. Drp1 affects synaptic formation, neurite outgrowth, and brain development [12].

Tumor necrosis factor receptor-associated protein 1 (TRAP1) was initially identified as an interacting protein that binds to the intracellular domain of TNF receptor 1 in vitro [21]. TRAP1 is a member of the heat shock protein 90 family and possesses ATPase activity [22]. Expression of TRAP1 is enhanced by a variety of stimuli, such as oxidative stress, hydroperoxidase stress, radioadaptive responses, and glucose deprivation [23]–[26]. TRAP1 has also been reported to play an important role in inhibiting cell death caused by reactive oxygen species (ROS) [27], [28]. Knockdown of TRAP1 using siRNA increases ROS accumulation, whereas TRAP1 overexpression decreases ROS production [29], [30]. Granzyme M, a serine protease capable of inducing apoptosis, can cleave TRAP1 to compromise ATPase activity and abolish its antagonistic function against ROS, resulting in ROS accumulation and cell death [29]. Thus, TRAP1 prevents damaged proteins from unfolding, refolds denatured proteins [31], and regulates ROS metabolism to antagonize ROS production, thereby maintaining the integrity of mitochondria under oxidative stress.

Although TRAP1 has various functions, as described above, we have shown that TRAP1 mediates TNF-alpha/TNF receptor 1 signaling to modulate N-cadherin expression and to regulate cell adhesion and synaptic morphology, which may be involved in the pathogenesis of major depression [32]. Furthermore, we also reported that TRAP1 regulates the unfolded protein response, and mediates the endoplasmic reticulum stress response, which is known to be concerned with cell death [33]. During the above experiments, we found that TRAP1 knockdown cells had tubular shaped mitochondria.

Here we focused on the relationship between TRAP1 and mitochondrial morphology. We report that TRAP1 regulates Drp1 and Mff expression and controls the changes to mitochondrial morphology.

## Materials and Methods

### Chemicals and Antibodies

The following antibodies were used: anti-β-tubulin mAb (Sigma-Aldrich, St. Louis, MO, USA), anti-glyceraldehyde 3-phosphate dehydrogenase (GAPDH) mAb (Santa Cruz Biotechnology, Santa Cruz, CA, USA), anti-hydroxyacyl-CoA dehydrogenase/3-ketoacyl-CoA thiolase/enoyl-CoA hydratase (trifunctional protein), alpha subunit (HADHA) pAb (Abcam, Cambridge, MA, USA), anti-TRAP1 mAb (BD Transduction Laboratories, Franklin Lakes, NJ, USA), anti-Mfn mAb (Abnova, Taipei, Taiwan), anti-Opa1 mAb (BD Transduction Laboratories), anti-Drp1 mAb (BD Transduction Laboratories), anti-Fis1 mAb (Enzo Life Sciences, Plymouth Meeting, PA, USA), anti-Parkin pAb (Abcam), HRP-conjugated anti-mouse IgG pAb (Cell Signaling, Beverly, MA, USA), HRP-conjugated anti-rabbit IgG pAb (Cell Signaling), Alexa 488-conjugated anti-mouse IgG pAb and Alexa 568-conjugated anti-rabbit IgG pAb (Invitrogen, Grand Island, NY, USA). Rabbit polyclonal antibodies against Mff were kindly provided by Dr. Paul E. Fraser (University of Toronto, Toronto, Canada). Rabbit polyclonal antibodies against MarchV were kindly provided by Dr. Shigehisa Hirose (Tokyo Institute of Technology, Yokohama, Japan). Rabbit polyclonal antibodies against MiD51/MIEF1 were kindly provided by Dr. Michael T. Ryan (La Trobe University, Melbourne, Australia). The chemical reagents used in these experiments were MG132 (Sigma-Aldrich).

### Construction of Expression Vectors

A plasmid containing a short hairpin RNA (shRNA) for human TRAP1 (shTRAP1) was derived from the eukaryotic expression vector, pcDNA6.2-GW/EmGFP-miR (Invitrogen). Human shTRAP1 was amplified with the following primer set:

5′-TGCTGATTTCAAACACTCCAGAACCAGTTTTGGCCACTGACTGACTGGTTCTGGTGTTTGAAAT-3′ (forward) and 5′-CCTGATTTCAAACACCAGAACCAGTCAGTCAGTGGCCAAAACTGGTTCTGGAGTGTTTGAAATC-3′ (reverse).

The plasmid, pcDNA6.2-GW/miR-neg, was used as a negative control.

A recombinant adenovirus expressing GFP, human TRAP1 with GFP fused to the C terminus, human Drp1 with HA-tag fused to the N terminus, human Mff and human Mff deleting C terminous which binds to mitochondria (MffΔC) were generated using the ViraPower Adenoviral Expression System (Invitrogen) according to the manufacturer’s instructions.

### Cell Culture and Infection

SH-SY5Y neuroblastoma cells (European Collection of Cell Culture) were cultured in Dulbecco’s modified Eagle’s medium (DMEM)/F12 (Invitrogen) containing 10% fetal bovine serum (FBS) at 37°C and 5% CO_2_. SH-SY5Y cells were transiently infected with adenovirus vectors for 48 h. The cells were incubated with MG132 (1 µM) for 16 h at 37°C.

KNS-42 glioma cells (kindly provided by Dr. Paul E. Fraser (University of Toronto, Toronto, Canada)) were cultured in DMEM (Invitrogen) containing 10% fetal bovine serum (FBS) at 37°C and 5% CO_2_.

### Western Blot Analysis

Cells were washed twice in phosphate buffered saline (PBS), harvested by scraping and resuspended in TNE buffer (10 mM Tris–HCl, pH 7.8, 1 mM EDTA and 150 mM NaCl) containing 1% (v/v) NP-40 and a protease inhibitor cocktail (Roche Diagnostics, Indianapolis, IN, USA). For mitochondrial cytoplasmic fractionation, cells were resuspended in homogenization buffer (250 mM sucrose, 20 mM HEPES pH 7.5, 10 mM KCl, 1.5 mM MgCl_2_, 1 mM EGTA and protease inhibitor cocktail). Cells were homogenized and the nuclear fraction was removed by centrifugation at 700×g for 10 minutes at 4°C. The post-nuclear supernatant was centrifuged at 5000×g for 10 minutes at 4°C to pellet the mitochondria. The supernatants were collected as the cytoplasmic fractions. The pellets were resuspended in TNE buffer containing 1% (v/v) NP-40 and protease inhibitor cocktail, and centrifuged at 15,000×g for 10 minutes to clear the mitochondrial fraction. Equal amounts of protein were subjected to sodium dodecyl sulphate-polyacrylamide gel electrophoresis (SDS–PAGE) and transferred to polyvinylidene difluoride (PVDF) membranes (Millipore, Billerica, MA, USA). The membranes were blocked with 5% (w/v) skim milk in PBS containing 0.05% Tween-20 (PBS-T) and then incubated with PBS-T containing primary antibody against TRAP1, Mfn, Opa1, Drp1, Mff, Fis1, HADHA or β-tubulin, followed by incubation with an HRP-conjugated secondary antibody. Signals were detected by enhanced chemiluminescence (ECL Western Blotting Detection System; GE Healthcare Life Sciences, Piscataway, NJ, USA).

### Immunocytochemistry

Cells were fixed with 4% paraformaldehyde in 0.1 M phosphate buffer (PB) for 30 minutes at room temperature. After 1 h incubation in blocking solution comprising 5% bovine serum albumin (BSA), 0.3% Triton X-100 in PBS, the cells were incubated in the same solution containing primary antibodies against TRAP1 or HADHA at 4°C for 24 h. The cells were then incubated in the secondary antibody solution containing 5% BSA and Alexa 568-conjugated anti-rabbit IgG antibody or Alexa 488-conjugated anti-mouse IgG antibody at room temperature (RT) for 1 h. The coverslips were mounted onto slides using Fluoromount (Diagnostic BioSystems, Pleasanton, CA, USA). Fluorescence images were acquired using a laser confocal microscope (LSM-510 UV/META, Carl Zeiss, Oberkochen, Germany).

### Electron Microscopy

SH-SY5Y cells and stable TRAP1 KD cells were fixed at RT for 1 h in 0.1 M PB containing 2.5% glutaraldehyde (GA) and 2% paraformaldehyde. Subsequently, the cells were post-fixed in 1% OsO_4_ at RT for 1 h, dehydrated in a graded ethanol series, and embedded in epoxy resin (Quetol 812; Nisshin EM Co., Tokyo, Japan). Areas containing cells with aggregates were block-mounted in epoxy resin by the direct epoxy-resin embedding method and cut into 90 nm sections. The sections were examined using an H-7100 electron microscope (Hitachi, Tokyo, Japan).

### RT-PCR

Total RNA was extracted from cultured cells using the High Pure RNA kit (Roche). RNA concentrations were determined spectrophotometrically at 260 nm. Each RNA was transcribed to cDNA using reverse transcription reagents (High-Capacity cDNA Reverse Transcription Kit; Applied Biosystems, Foster City, CA, USA) according to the manufacturer’s instructions. Real-time RT-PCR was performed on a thermocycler (7900HT Sequence Detection Systems; Applied Biosystems) with nuclear stain reagents (SYBR Green; Applied Biosystems) according to the manufacturer’s instructions. The amplification conditions for semiquantitative RT-PCR analysis were as follows: an initial denaturation step (95°C for 10 min), followed by 40 cycles of 95°C for 15 s and 60°C for 1 min. Amplification of PCR products was measured by fluorescence associated with the binding of double-stranded DNA to the SYBR green dye in the reaction mixture. The oligonucleotide sequences used for PCR were as follows: for Drp1, forward: 5′-CACCCGGAGACCTCTCATTC-3′ and reverse: 5′-CCCCATTCTTCTGCTTCCAC-3′; for Mff, forward: 5′-CCAAACGCTGACCTGGAAC-3′ and reverse: 5′-TTTCCTGCTACAACAATCCTCTCC-3′; and for β-actin, forward: 5′-GCACTCTTCCAGCCTTCCTT-3′ and reverse: 5′-CGTACAGGTCTTTGCGGATG-3′.

### Preparation of siRNAs and Transfection

Small interfering RNAs (siRNAs) specific to human TRAP1 (Mission siRNA, Hs_TRAP1_9215, Sigma-Aldrich) were purchased. Scrambled siRNAs for the human genome (Mission_SIC-001, Sigma-Aldrich) were used as controls. siRNA transfection was performed with Lipofectamine RNAiMAX (Invitrogen) according to the manufacturer’s protocol. SH-SY5Y cells were transfected with 100 nM siRNA against TRAP1 or scrambled siRNA and then incubated for 48 h. KNS cells were transfected with 25 nM siRNA against TRAP1 or scrambled siRNA and then incubated for 48 h.

## Results

### Abnormal Morphology of Mitochondria is Observed in Stable TRAP1 KD Cells

**Figure 1:**
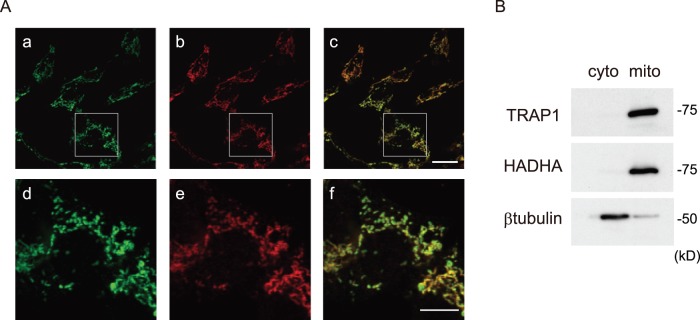
Localization of TRAP1 in SH-SY5Y cells. A, SH-SY5Y cells were stained with anti-TRAP1 and anti-HADHA antibodies (mitochondrial marker) (a,d, TRAP1, green; b,e, HADHA, red; c,f, overlapping, yellow) and observed under a confocal microscope as described in the Materials and Methods. Upper panels show low magnification images. Lower panels show higher magnification images of part of each low magnification image (white windows). B, Equal amounts of lysates from cytoplasm (cyto) and mitochondria (mito) were analyzed by western blotting using anti-TRAP1 (upper panels), anti-HADHA (middle panel) or β-tubulin antibodies (lower panels). Scale bars, 20 µm (A, upper), 10 µm (A, lower).

**Figure 2:**
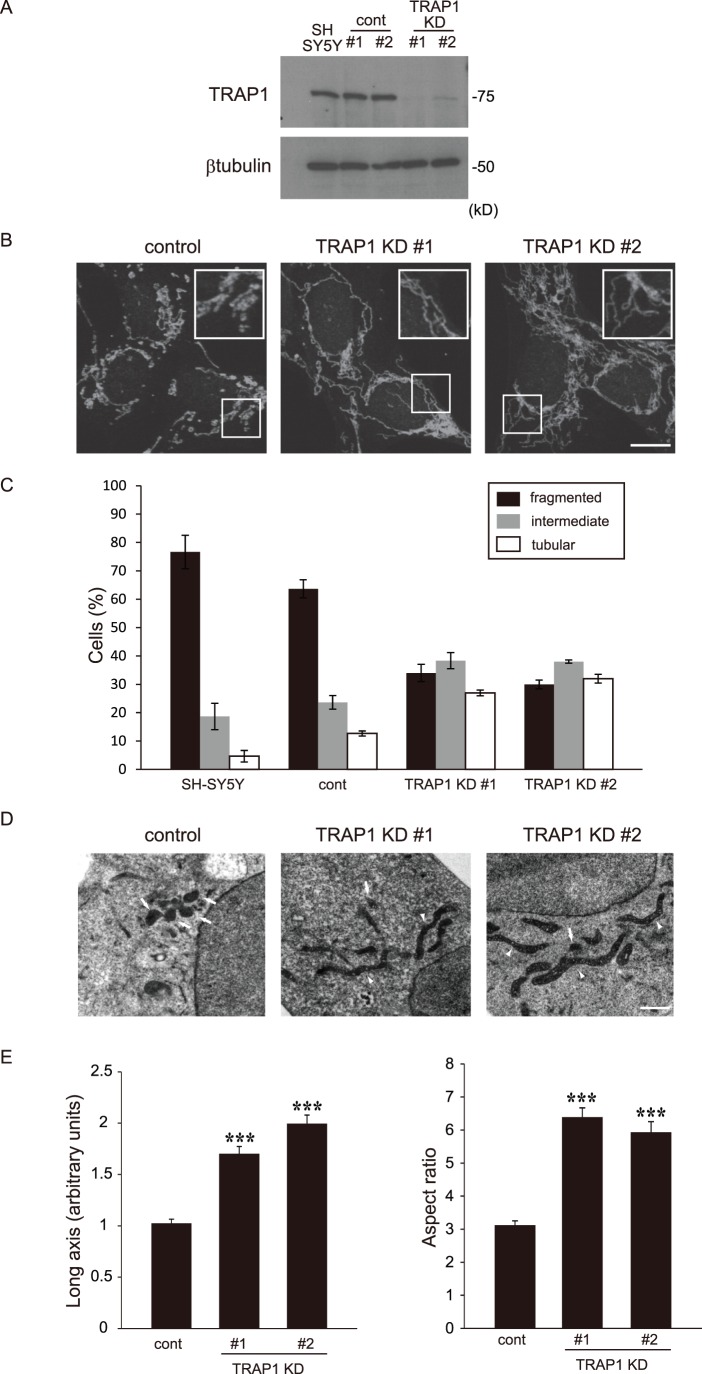
The effect of KD on TRAP1 expression and mitochondrial morphology. A, Specific reduction of TRAP1 levels in the clone stably expressing human TRAP1 shRNA, as shown by western blotting. Total proteins isolated from normal SH-SY5Y cells, control shRNA-expressing cells (cont) and the TRAP1 shRNA-expressing SH-SY5Y cells (TRAP1 KD) were blotted using antibodies against TRAP1 and β-tubulin. B, Cells were stained with an antibody against the mitochondrial marker, HADHA. High magnification images of a part of each photograph are shown in the white windows. C, Percentages of cells with the indicated mitochondrial morphologies in normal SH-SY5Y cells, control and TRAP1 KD cells. Data were obtained from at least three independent experiments and represent the means ± s.e.m. D, Representative electron micrographs of control and stable TRAP1 KD cells. Arrows indicate examples of fragmented mitochondria, and arrowheads indicate examples of tubular mitochondria. E, Quantification of mitochondrial length in each cell. The graph on the left shows the length of the mitochondrial long axis. The graph on the right shows the ratio of the lengths of the mitochondrial long axis and short axis (aspect ratio). The data are expressed as the means ± s.e.m. of 126 mitochondria (13 cells, control), 175 mitochondria (11 cells, KD #1), and 117 mitochondria (11 cells, KD #2). The asterisks on each bar indicate statistical significance among the groups (long axis: one-way ANOVA, *P* = 9.06×10^−20^, post hoc Games-Howell test, ***P<0.001; aspect ratio: one-way ANOVA, *P* = 1.55×10^−17^, post hoc Games-Howell test, ***P<0.001). Scale bars, 10 µm (B), 1 µm (D).

It has been reported that TRAP1 is a mitochondrial protein [22], [34]. Here, we observed the effect of TRAP1 on mitochondrial morphology in SH-SY5Y cells. Immunofluorescence analysis with a TRAP1 antibody showed colocalization with HADHA, a mitochondrial marker, with large fragmented shape (Fig. 1A). Moreover, TRAP1 expression was strongly detected in the mitochondrial fraction, but was only very weakly detected in the cytoplasmic fraction by western blot analysis (Fig. 1B). To investigate the role of TRAP1 in mitochondrial morphology, SH-SY5Y cells were transfected with shRNA of TRAP1, to obtain cells with a stable knockdown (KD) of endogenous TRAP1 expression. The two pairs of shRNA were similarly effective. Results are shown with #1 and #2, which reduced the protein levels of TRAP1 by almost 100% (Fig. 2A). Cells stably knocked-down for TRAP1 showed no change in total cell morphology under normal conditions (data not shown). We studied whether TRAP1 expression levels affected mitochondrial morphology. Immunofluorescent microscopy showed that control SH-SY5Y cells showed fragmented mitochondria under our experimental conditions. On the other hand, tubular shaped mitochondria were observed in stable TRAP1 KD cells (Fig. 2B). Mitochondrial morphology was also observed by electron microscopy (Fig. 2D). Tubular mitochondria were observed in stable TRAP1 KD cells (arrowhead), although there were many fragmented mitochondria in control cells (arrow). There was a notable increase in mitochondrial connectivity in the stable TRAP1 KD cells (Fig. 2C,E). The number of fragmented mitochondria decreased in stable TRAP1 KD cells.

### TRAP1 has no Effect on Mitochondrial Fusion Proteins

**Figure 3:**
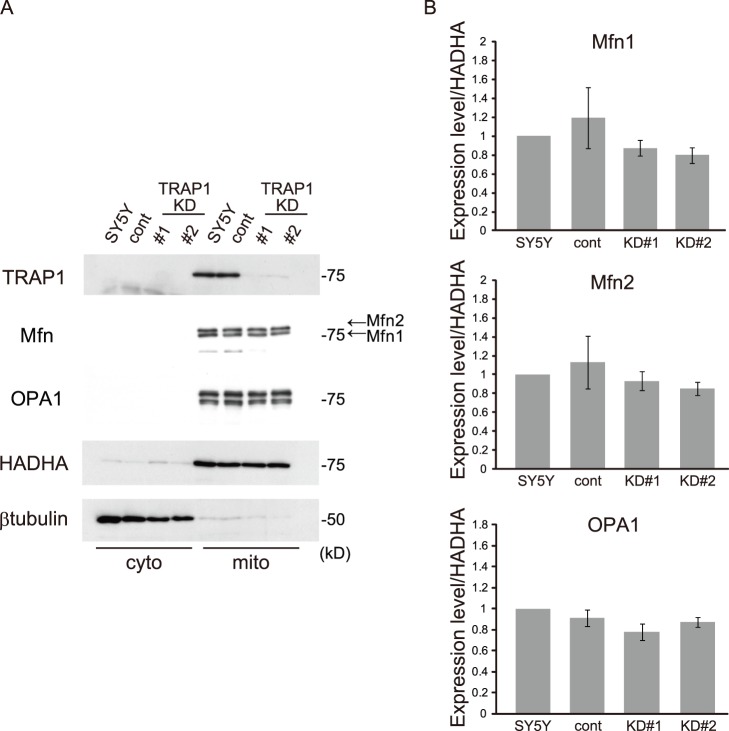
The effect of TRAP1 on the expression levels of mitochondrial fusion proteins. A, Cells were fractionated into cytoplasm (cyto) and mitochondrial (mito) fractions, which were analyzed by western blotting using antibodies against mitofusin (Mfn), OPA1, TRAP1, HADHA (mitochondrial marker) and β-tubulin (cytoplasmic marker). B, Quantification of the expression levels of Mfn1/2 and OPA1. These data were obtained by densitometric analysis of each band on the western blots, and the data are expressed as the means ± s.e.m. of at least four independent experiments. β-tubulin (cyto) and HADHA (mito) were used as internal controls. One-way ANOVA showed no statistically significant difference between the groups (Mfn1, *P* = 0.414; Mfn2, *P* = 0.636; OPA1, *P* = 0.093).

To identify which molecules relate to changes in mitochondrial morphology in stable TRAP1 KD cells, first we searched for changes in mitochondrial fusion proteins by western blot analysis. As previously reported, both Mfn1/2 and OPA1 were expressed in the mitochondrial fraction (Fig. 3A). Both TRAP1 KD cells did not change expression levels of the fusion proteins Mfn1, Mfn2 and OPA1 (Fig. 3B).

### Effect of TRAP1 KD on the Expression of Mitochondrial Fission Proteins

**Figure 4:**
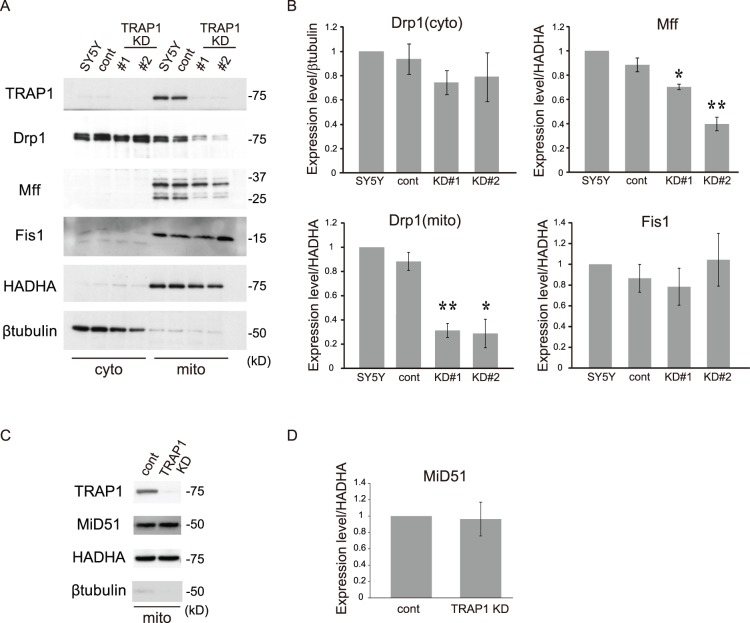
The effect of TRAP1 on the expression levels of mitochondrial fission proteins. A, A, The expression levels of Drp1, Mff and Fis1 were analyzed by western blotting. B, Quantification of the expression levels of Drp1, Mff and Fis1. The conventions are the same as those shown in Fig. 3. The asterisks on each bar indicate statistical significance between the group and normal SH-SY5Y cells (Drp1 (cyto): one-way ANOVA, *P* = 2.02×10^−5^, post hoc Games-Howell test, *P<0.05, **P<0.01; Mff: one-way ANOVA, *P* = 1.53×10^−6^, post hoc Games-Howell test, *P<0.05, **P<0.01; Fis1: one-way ANOVA, *P* = 0.684). C, Mitochondrial fractions from cells were blotted with antibodies against TRAP1, MiD51/MIEF1, HADHA and β-tubulin. D, Quantification of the expression level of MiD51/MIEF1. Data are expressed as the means ± s.e.m. of seven independent experiments. Welch’s t-test showed no statistical significance (*P* = 0.863).

Next, we observed the expression levels of the mitochondrial fission proteins in stable TRAP1 KD cells. Similar to previous reports, Drp1 was expressed mainly in the cytoplasmic fraction and small amounts were expressed in the mitochondrial fraction. Other molecules, namely, Mff and Fis1, were expressed mainly in the mitochondrial fraction (Fig. 4A). In stable TRAP1 KD cells, significant decreases in mitochondrial Drp1 and Mff levels were observed in the mitochondrial fraction, although there were no changes in the expression levels of cytoplasmic Drp1 and Fis1 (Fig. 4A,B). We also examined the expression levels of the novel factor MiD51/MIEF1 [35], [36]. There was no change in the expression level of MiD51/MIEF1 in stable TRAP1 KD cells (Fig. 4C,D). These findings indicate that TRAP1 controls mitochondrial morphology through regulation of Drp1 and Mff.

### TRAP1 Overexpression Rescues Mitochondrial Morphology and the Down-regulation of Mitochondrial Drp1 and Mff in Stable TRAP1 KD Cells

**Figure 5:**
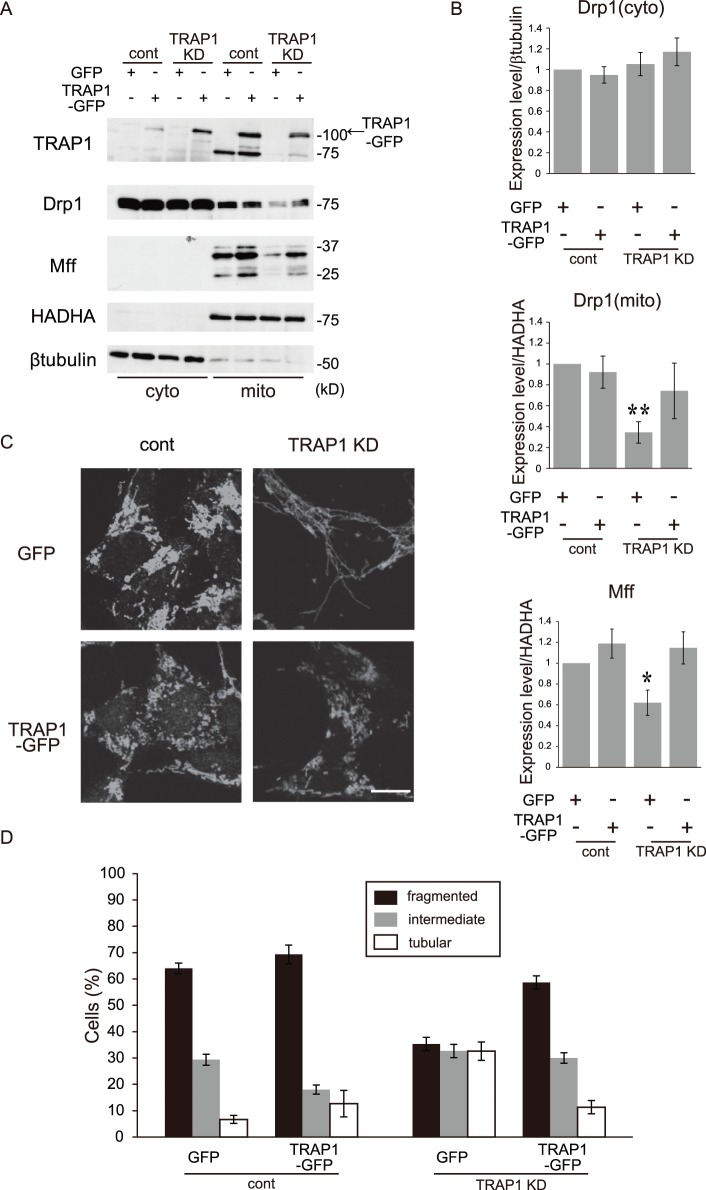
Overexpression of TRAP1 affects mitochondrial morphology and the expression level of mitochondrial fission proteins. A, Cells infected with the GFP or TRAP1-GFP adenoviruses were fractionated into cytoplasmic (cyto) and mitochondrial (mito) fractions and blotted with antibodies against TRAP1, Drp1, Mff, HADHA and β-tubulin. B, Quantification of the results shown in panel A. The data are expressed as the means ± s.e.m. of at least six independent experiments. The conventions are same as those shown in Fig. 3. The asterisks on each bar indicate statistical significance between the group and the normal SH-SY5Y cells (Drp1 (cyto): One-way ANOVA, *P* = 0.413; Drp1 (mito): one-way ANOVA, *P* = 0.039, post hoc Games-Howell test, **P<0.01; Mff: one-way ANOVA, *P* = 0.011, post hoc Games-Howell test, *P<0.05). C, Cells treated as in A were analyzed by immunofluorescence microscopy using anti-HADHA. D, Percentages of cells with the indicated mitochondrial morphology in C. Data were collected from six independent experiments and represent means ± s.e.m. Scale bar, 10 µm (C).

To confirm the effect of TRAP1 on mitochondrial morphology and on the expression levels of mitochondrial fission proteins, we overexpressed TRAP1-GFP in stable TRAP1 KD cells. Using western blot analysis, we confirmed the overexpression of the full-length human TRAP1 protein tagged with GFP. Expression levels of TRAP1 protein in these cells recovered to the same level as that in control cells (Fig. 5A). As shown in Fig. 4, mitochondrial Drp1 and Mff expression significantly decreased in stable TRAP1 KD cells compared with control cells (Fig. 5A,B). The reduced levels of mitochondrial Drp1 and Mff found in stable TRAP1-KD cells were significantly rescued in stable TRAP1 KD cells following recovery of TRAP1 expression (Fig. 5A,B). In control cells, expression levels of mitochondrial Drp1 and Mff remained unchanged following overexpression of TRAP1-EGFP. We also observed the effect of recovered TRAP1 expression on mitochondrial morphology using immunocytochemistry (Fig. 5C,D). TRAP1 overexpression induced fragmentation of mitochondria and decreased the number of tubular mitochondria in stable TRAP1 KD cells, confirming the regulation of mitochondrial morphology through Drp1 and Mff expression by TRAP1.

### Overexpression of Mff, but not Drp1, Induces Mitochondrial Fragmentation in Stable TRAP1 KD Cells

**Figure 6:**
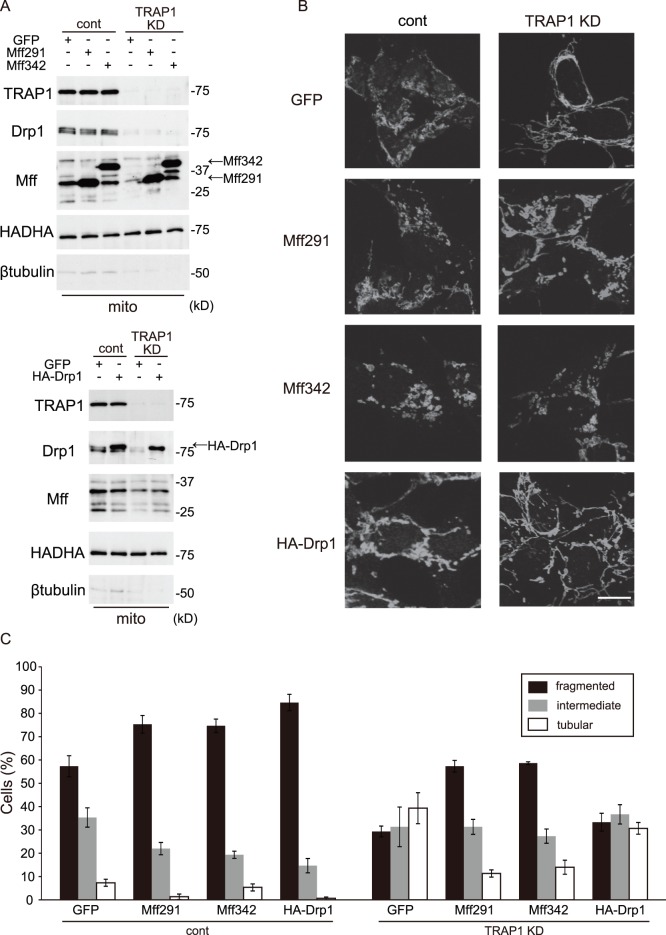
The effect of Drp1 and Mff overexpression on mitochondrial morphology. A, Mitochondrial fractions (mito) from cells infected with GFP, Mff291, Mff342 or HA-Drp1 were blotted with antibodies against TRAP1, Drp1, Mff, HADHA and β-tubulin. B, Cells treated as in A were analyzed by immunofluorescence microscopy using anti-HADHA antibody. C, Percentages of cells with the indicated mitochondrial morphology in B. Data were collected from three independent experiments and represent means ± s.e.m. Scale bar, 10 µm (B).

It has been reported that Mff is able to multimerize [2], and regulates the function of Drp1 in mitochondrial fission [5]. To elucidate the relationship between TRAP1 and fission proteins, we overexpressed HA-Drp1 or Mff in stable TRAP1 KD cells. Mff has at least nine different isoforms, as deduced from the gene sequence, and four bands were detected by western blot analysis. We sub-cloned two major Mff isoforms, Mff291 (deduced amino acid number 291) and Mff342 (deduced amino acid number 342), into adenovirus vectors. We confirmed that the overexpression of Mff or Drp1 tagged with HA in these cells recovered by more than that in control cells using western blot analysis (Fig. 6A). Using immunocytochemistry, stable TRAP1 KD cells, Mff291 and Mff342 overexpression induced fragmentation of mitochondria compared with GFP transfection and reduced the number of tubular mitochondria when compared with GFP transfection. In addition, overexpression of HA-Drp1 did not change mitochondrial morphology when compared to GFP transfection in stable TRAP1 KD cells (Fig. 6B,C).

### MffΔC does not Affect Mitochondrial Morphology

**Figure 7:**
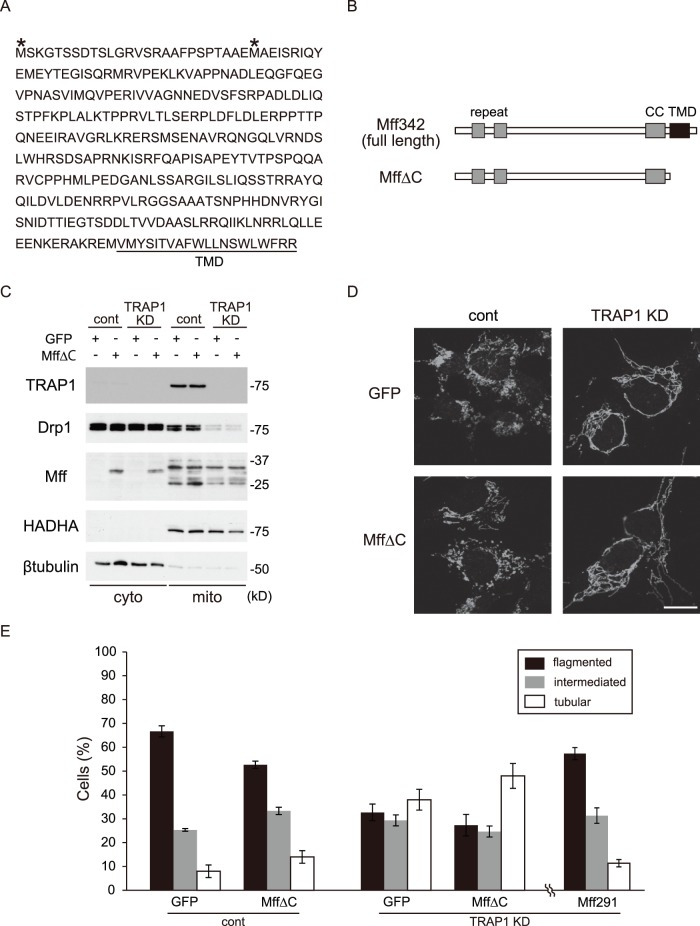
MffΔC does not rescue the abnormal mitochondrial morphology. A, Alignment of Mff amino acid sequence. Asterisks shows alternative translation-start sites. The transmembrane domain (TMD) at the carboxy-terminus is underlined. B, Schematic drawing of Mff342 and MffΔC. C, Cells infected with GFP or MffΔC were fractionated into cytoplasmic (cyto) and mitochondrial (mito) fractions and blotted with antibodies against TRAP1, Drp1, Mff, HADHA and β-tubulin. D, Cells treated as in C were analyzed by immunofluorescence microscopy using anti-HADHA. E, Percentages of cells with the indicated mitochondrial morphology in D. Data were collected from three independent experiments and represent means ± s.e.m. Scale bar, 10 µm (D).

Mff has a transmembrane domain on the carboxy-terminus, which determines mitochondrial localization [2] (Fig. 7A,B). To confirm the importance of the mitochondrial localization of Mff in regulating mitochondrial morphology, we used Mff**Δ**C vector. Mff**Δ**C vector is derived from Mff342 vector and lacks a transmembrane domain (Fig. 7B). The expression pattern of Mff**Δ**C is shown in Fig. 7C. Mff**Δ**C did not affect mitochondrial morphology in control cells. In TRAP1 KD cells, there was no change in the ratio of mitochondrial morphology between GFP and Mff**Δ**C-transfected cells, which was in contrast to Mff291 transfection (Fig. 7D,E). These results suggest that Mff in mitochondria is important for Drp1 function, and that it regulates mitochondrial morphology.

### TRAP1 Regulates the Expression Levels of Drp1 and Mff via a Nontranscriptional Mechanism

**Figure 8:**
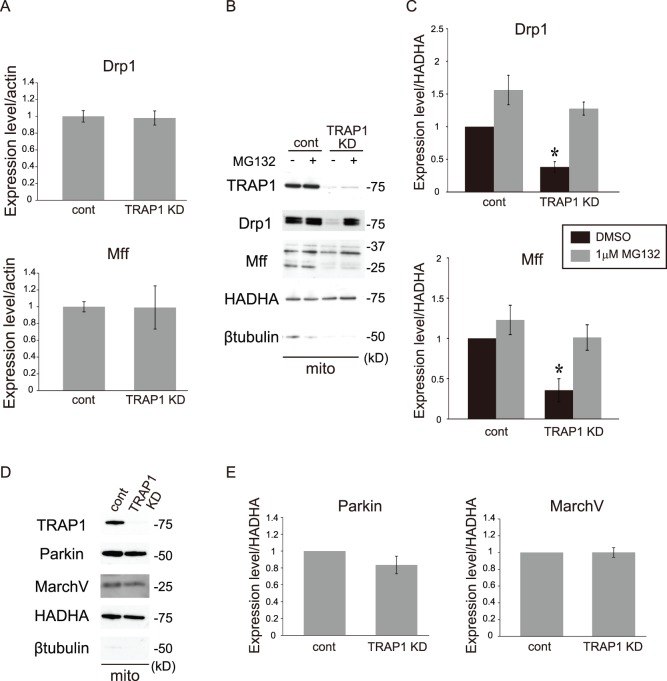
MG132 treatment affects the expression levels of Drp1 and Mff in TRAP1 KD cells. A, Quantitative RT-PCR of Drp1 and Mff mRNA levels in TRAP1 knockdown cells. Actin mRNA was used as an internal control. Data represent the means ± s.e.m. (Drp1: *P* = 0.863; Mff: *P* = 0.975, vs. control, Welch’s t-test) (n = 3). B, Cells treated with 1 µM MG132 were fractionated into mitochondrial (mito) fractions and blotted with antibodies against TRAP1, Drp1, Mff, HADHA and β-tubulin. C, Quantification of the results shown in panel B. The data are expressed as the means ± s.e.m. of at least six independent experiments. The asterisks on each bar indicate statistically significant differences between the group and the normal SH-SY5Y cells (Drp1: one-way ANOVA, *P* = 2.19×10^−5^, post hoc Games-Howell test, *P<0.05; Mff: one-way ANOVA, *P* = 1.94×10^−4^, post hoc Games-Howell test, *P<0.05). D, Cells were fractionated into mitochondrial (mito) fractions and blotted with antibodies against TRAP1, Parkin, MarchV, HADHA and β-tubulin. E, Quantification of the results shown in panel D. Data represent the means ± s.e.m. (Parkin: *P* = 0.159; Mff: *P* = 0.994, vs. control, Welch’s t-test) (Parkin: n = 8; MarchV: n = 3).

We next examined whether TRAP1 regulates Drp1 and Mff at the mRNA level in control cells and stable TRAP1 KD cells (Fig. 8A). Drp1 and Mff transcription was unchanged in stable TRAP1 KD cells. We also observed the effect of MG132, a proteasome inhibitor, on Drp1 and Mff expression (Fig. 8B,C). MG132 treatment resulted in significant upregulation of Drp1 and Mff protein levels. These results suggest that the regulation of Drp1 and Mff expression by TRAP1 is not transcriptional. Because Drp1 is ubiquitinated, and the Drp1 protein level has been reported to be regulated by two E3 ubiquitin ligases, Parkin and MarchV [37]–[40], we examined the levels of these ligases by western blotting. In stable TRAP1 KD cells, Parkin and MarchV levels were not changed significantly, even though Parkin expression tended to decrease in stable TRAP1 KD cells (Fig. 8D,E).

### Abnormal Morphology of Mitochondria and Downregulation of Drp1 and Mff in Transient TRAP1 KD Cells (SH-SY5Y Cells)

**Figure 9:**
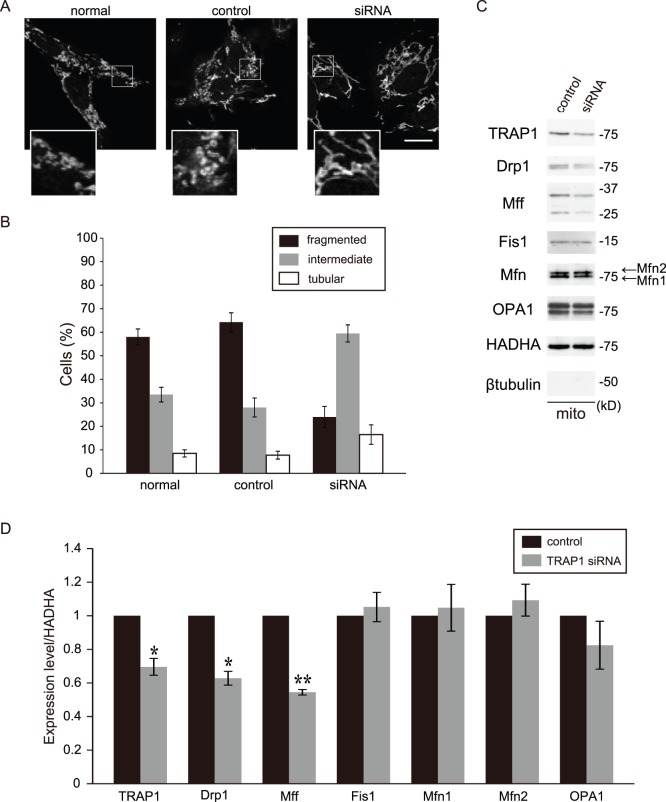
The effect of transient TRAP1 KD on mitochondrial morphology in SH-SY5Y cells. A, Cells were analyzed by immunofluorescence microscopy using anti-HADHA antibody. High magnification images of a part of each photograph are shown in the white windows (normal, untreated; control, scrambled siRNA; siRNA, siRNA to TRAP1). B, Percentages of cells with the indicated mitochondrial morphologies in normal SH-SY5Y cells, control and TRAP1 KD cells. C, Cells were fractionated into mitochondrial (mito) fractions and blotted with antibodies against TRAP1, Drp1, Mff, fis1, Mfn1/2, OPA1, HADHA and β-tubulin. D, Quantification of the results shown in panel C. Data are expressed as the means ± s.e.m. of at least three independent experiments. The conventions are the same as those shown in Fig. 3. The asterisks on each bar indicate statistical significance (TRAP1: *P* = 0.025, Drp1: *P* = 0.012, Mff: *P* = 0.001, Fis1: *P* = 0.614, Mfn1: *P* = 0.764, Mfn2: *P* = 0.432, OPA1: *P* = 0.343, vs. control, Welch’s t-test, *P<0.05, **P<0.01). Scale bar, 10 µm (A).

We also observed the effects of transient TRAP1 KD using siRNA transfection in SH-SY5Y cells. As in stable TRAP1 KD cells, abnormal mitochondrial morphology was observed in transient TRAP1 KD cells (Fig. 9A,B). The siRNA knocked the TRAP1 protein level down to <70% compared with the control level. Transient TRAP1 KD in SH-SY5Y cells also decreased Drp1 and Mff levels in the mitochondrial fraction (Fig. 9C,D).

### Dysregulation of Mitochondrial Morphology and Fission Proteins Expression in TRAP1 KD Cells (KNS Cells)

**Figure 10:**
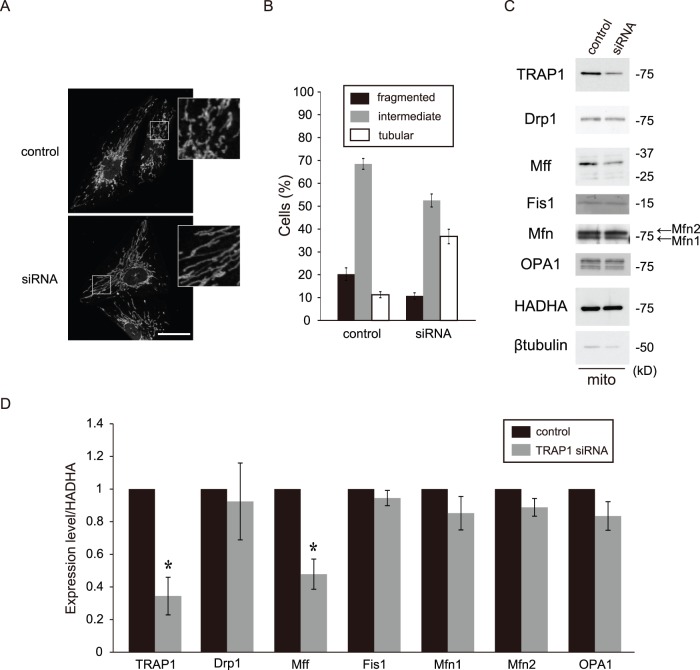
Transient TRAP1 KD induces abnormal mitochondrial morphology and a strong decrease in Drp1 level in KNS cells. A, Cells were stained with an anti-HADHA antibody and analyzed by immunofluorescence microscopy. High magnification images of a part of each photograph are shown in the white windows (control, scrambled siRNA; siRNA, siRNA to TRAP1). B, Percentages of cells with the indicated mitochondrial morphologies in control and TRAP1 KD cells. C, Mitochondrial fractions (mito) from each cell group were blotted with antibodies against TRAP1, Drp1, Mff, fis1, Mfn1/2, OPA1, HADHA and β-tubulin. D, Quantification of the results shown in panel C. The data are expressed as the means ± s.e.m. of at least three independent experiments. The conventions are the same as those shown in Fig. 3. The asterisks on each bar indicate statistical significance (TRAP1: *P* = 0.030, Drp1: *P* = 0.778, Mff: *P* = 0.030, Fis1: *P* = 0.355, Mfn1: *P* = 0.286, Mfn2: *P* = 0.173, OPA1: *P* = 0.199, vs. control, Welch’s t-test, *P<0.05). Scale bar, 20 µm (A).

To obtain definitive evidence for TRAP1’s function in the regulation of mitochondrial morphology, we investigated the effect of transient TRAP1 KD by siRNA in KNS-42 cells. Tubular mitochondria were observed in transient TRAP1 KD cells (Fig. 10A,B). A significant decrease in mitochondrial Mff was observed in the mitochondrial fraction, although there were no changes in the expression levels of Drp1, Fis1 and fusion proteins (Fig. 10C,D).

## Discussion

### Effect of TRAP1 on Mitochondrial Fusion and Fission Proteins

In this report, silencing of TRAP1 shifted the balance towards a fusion phenotype, characterized by extensive tubular mitochondria. Therefore, to clarify whether TRAP1 affected mitochondrial fusion or fission, we characterized the expression levels of fusion and fission proteins in TRAP1 KD cells by western blotting. Our results showed that TRAP1 did not affect the mitochondrial fusion proteins Mfn1/2 and OPA1. In contrast, levels of the fission proteins Drp1 and Mff were significantly decreased in stable TRAP1 KD cells via a nontranscriptional mechanism, although other fission proteins, namely, Fis1 and MiD51/MIEF1, were not affected. Previous reports have suggested that the fusion/fission balance is maintained reciprocally [12], [41]. For example, Drp1 knock out results in downregulation of fusion proteins, such as OPA1 and Mfn, in addition to inhibition of Drp1 expression [12]. In our results, TRAP1 KD tended to decrease Mfn and OPA1 levels, although the changes were not significant. These results suggest that TRAP1 has stronger effects on fission proteins than fusion proteins. Compared with the results in TRAP1 KD SH-SY5Y cells, we detected no change in Drp1 expression in TRAP1 KD KNS-42 cells, although Mff downregulation and abnormal mitochondrial morphology were observed in KNS-42 cells. These results suggest that Mff is a common protein in the mechanisms by which TRAP1 regulates mitochondrial morphology, regardless of cell type.

The effect of TRAP1 KD on mitochondrial morphology observed in our study was somewhat different from that reported in a previous study [42]. This could be due to different culture conditions, differences in cell cycle stages, or different providers of SH-SY5Y cells. There is a relationship between mitochondrial morphology and cell cycle progression [43]–[45]. Mitochondria in cells in interphase are long tubular network structures that are fragmented in the early mitotic phase, and the filamentous network structures are subsequently reformed in the daughter cells. In addition to this, we observed mitochondrial morphology using new SH-SY5Y cell purchased from the provider described in the previous report, furthermore, using the other cell line KNS-42. However, the results were the same.

### Regulation of Drp1 and Mff Expression in Stable TRAP1 KD Cells

Because Drp1 and Mff levels decreased severely following reduced expression of TRAP1, it is thought that TRAP1 affected Drp1 and Mff localization to mitochondria, or their expression levels. In our report, Drp1 and Mff expression were nontranscriptionally regulated by TRAP1. Mff has deduced several isoforms, and is detected as four major bands by western blotting [2], [5]. Our result showed that the level of the longer isoform of Mff is increased by MG132 treatment in TRAP1 KD cells. The longer isoform of Mff is expressed in brain or neuronal cells [2]. Therefore, each isoform may be regulated by different mechanisms. There are two possible mechanisms for regulating protein expression. First, TRAP1 may control the ubiquitination of Drp1 and/or Mff directly, consequently leading to degradation of the protein. A recent report suggested that the proteasome is localized to the mitochondrial outer membrane, and that it degrades mitochondrial outer and inner membrane proteins [46]. Expression levels of Drp1 are regulated by ubiquitination, and this ubiquitination affects mitochondrial dynamics. For example, Parkin and MarchV E3 ubiquitin ligases regulate Drp1 ubiquitination [37]–[40], [47]. However, we did not detect changes in the expression levels of Parkin and MarchV. Another ubiquitination pathway may contribute to Drp1 and Mff expression. In fact, TRAP1 controls the cellular ubiquitination and expression levels of mitochondrial proteins [48]. Second, SUMOylation may be involved in the decrease of Drp1 and Mff in stable TRAP1 KD cells. SUMOylation is essential for the maintenance of mitochondrial morphology and function. Drp1 is SUMOylated, and its SUMOylation causes stabilization, resulting in the upregulation of the protein [49]–[51].

Fission of mitochondria is induced by overexpression of Mff, even when expression of TRAP1 is low, but overexpression of Mff**Δ**C did not affect the morphology of mitochondria. However, Drp1 overexpression did not affect mitochondrial morphology when Mff expression was low in stable TRAP1 KD cells. These results suggest that mitochondrial Mff is important for Drp1 function, as reported previously [5].

### Possible Role of TRAP1 for Mitochondrial Function

It has been reported that TRAP1 controls a variety of mitochondrial functions and is protective against many stressful stimuli. TRAP1 overexpression decreases ROS production, free radical generation and lipid peroxidation, and preserves mitochondrial membrane potential, ATP levels and complex IV activity under various stressful conditions [42], [52]–[54]. Mitochondrial morphological changes are essential for the maintenance of normal mitochondrial function [1], [55], [56]. Our present results suggest that TRAP1 controls these mitochondrial functions by regulating mitochondrial morphology. As described above, it is known that there is a relationship between mitochondrial morphology and cell cycle progression [43]–[45].

### TRAP1 Function in Neuronal Systems

Mitochondrial abnormalities have been documented in some of the major neurodegenerative diseases including Parkinson’s disease, Alzheimer’s disease, amyotrophic lateral sclerosis, and Huntington’s disease [57]–[59]. Recently, TRAP1 was reported as a downstream phosphorylation target of PTEN-induced kinase 1 (PINK1), a gene involved in the onset of Parkinson’s disease, in rat and human cell lines [60]. PINK1 also regulates the expression levels of TRAP1 [61]. It has been shown that factors contributing to neurodegenerative diseases, such as PINK1 and Parkin, affect both mitochondrial fusion and fission depending on the cell type and culture conditions [62]–[67]. TRAP1 may contribute to the pathogenesis of neurodegenerative diseases, in particular, Parkinson’s disease.

Normal mitochondrial function is also required for the development of neurons and the brain. A newborn girl with a dominant-negative allele for Drp1 shows abnormal brain development [68]. Moreover, Drp1-deficient mice show abnormal synaptic formation and brain development [12], [69]. Mitochondrial transport to peripheral neurites is essential for neurite outgrowth, synaptic formation and synaptic function [70]. TRAP1 may be associated with neuron and brain development.

Thus, it is plausible that TRAP1 controls mitochondrial fusion/fission balance, thereby regulating mitochondrial/cellular function. Moreover, TRAP1 may contribute to the regulation of normal brain development and/or the onset of neurodegenerative diseases.

## Acknowledgments

We thank Dr. Paul E. Fraser for the gift of the anti-Mff antibody and Mff**Δ**C expression vector, Dr. Shigehisa Hirose for the gift of the anti-MarchV antibody and Dr. Michael T. Ryan for the gift of the anti-MiD51/MIEF1 antibody. We also thank Mrs. E. Moriya, Mrs. Y. Ohashi and Mrs. K. Ueno for preparing this manuscript.
